# Unrecognized Cell Torpidity as a Risk Factor in Elective Plastic Surgery

**DOI:** 10.1097/GOX.0000000000001727

**Published:** 2018-03-12

**Authors:** Giovanni Nicoletti, Marco Saler, Marco Mario Tresoldi, Silvia Scevola, Angela Faga

**Affiliations:** From the * Plastic and Reconstructive Surgery, Department of Clinical Surgical Diagnostic and Pediatric Sciences, University of Pavia, Pavia, Italy; † Advanced Technologies for Regenerative Medicine and Inductive Surgery Research Center, University of Pavia, Pavia, Italy; ‡ Plastic and Reconstructive Surgery Unit, Maugeri Clinical Scientific Institutes, Pavia, Italy; and § Department of Molecular Medicine, University of Pavia, Pavia, Italy.

## Abstract

Supplemental Digital Content is available in the text.

In our laboratories, we are carrying out investigations on human fibroblasts under different experimental conditions.^1^ The fibroblasts are derived from skin samples harvested during reduction mammaplasties (protocol approved by the Hospital Ethical Committee, number 2064). The latter procedures are performed using our original variation of the Lejour breast reduction technique,^2^ whose main complications include moderate wound dehiscence and limited fat necrosis with an overall rate of 10%. Other complications include postoperative hematoma requiring surgical revision (4%), temporary nipple sensation loss (2%), and major fat necrosis with wide wound dehiscence (2%), the latter being reported only in patients with large adipose breasts.

A few months ago, we performed a breast reduction in a formerly obese nonsmoker patient, in apparently healthy conditions, complaining of chronic cervical and dorsal pain related to her breast hypertrophy.^3^ The patient’s body mass index at the time of the operation was 34; her medical history included long-standing hypothyroidism successfully treated with thyroid hormone replacement therapy and an anxious syndrome treated with citalopram, a selective serotonin reuptake inhibitor. Due to a relevant breast asymmetry, the reduction accounted for 1,169.5 g in the right breast and 674 g in the left one, and the reduction of the distance between the clavicular notch and the nipple was 17 cm on the right and 13 cm on the left.

A slow onset venous congestion in the nipple-areola complex yielding to a bilateral partial nipple-areola complex necrosis and a major wound dehiscence with fat necrosis in both breasts complicated the postoperative course. The patient underwent surgical debridement and a combination of local treatments including negative pressure therapy, hyaluronan felt application, iodine powder–based advanced dressing, and an *Equisetum arvense*- and collagen-based ointment that eventually allowed a complete wound healing in 10 weeks.

Interestingly, the skin samples harvested from this patient and cultured in Dulbecco’s Modified Eagle Medium (DMEM) enriched with fetal bovine serum, penicillin, streptomycin, gentamicin, and L-glutamine^4^ displayed an unusually torpid fibroblast growth trend in comparison with the cultures derived from other patients (Figs. 1, 2; **see figure, Supplemental Digital Content 1**, which displays morphology of the patient’s fibroblast culture versus a standard control at 24, 72, and 120 hours. Culture medium: DMEM. Scale bar: 10 μm, http://links.lww.com/PRSGO/A719). The viable cell counts in the fibroblast culture from this patient at 24, 48, 72, 96, and 120 hours reported markedly lower values than the counts at the same times in a standard control culture.

**Fig. 1.**
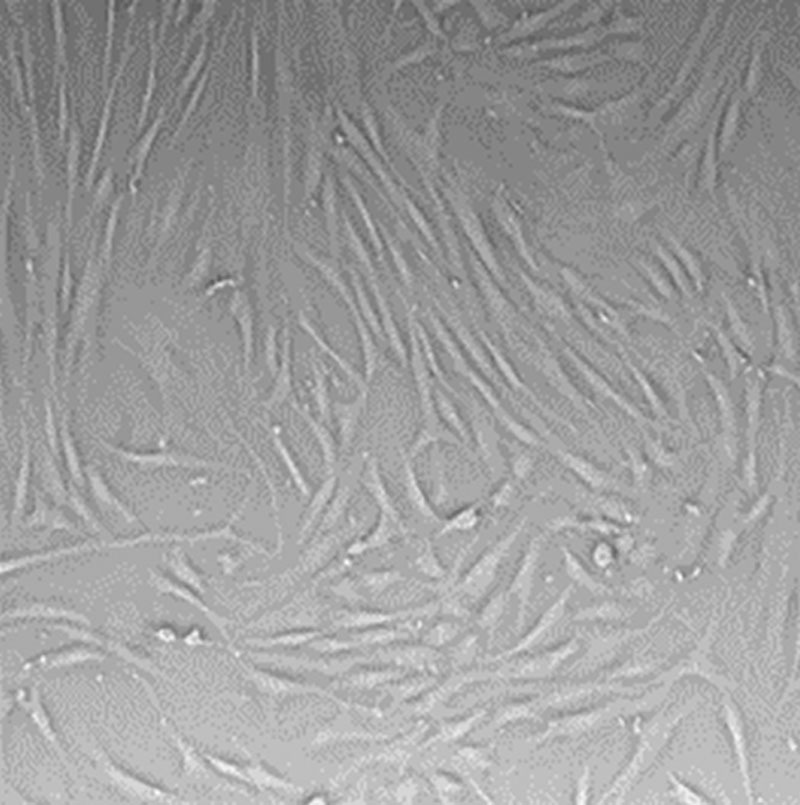
Morphology of a standard control fibroblast culture at 96 hours. Culture medium: DMEM.

**Fig. 2.**
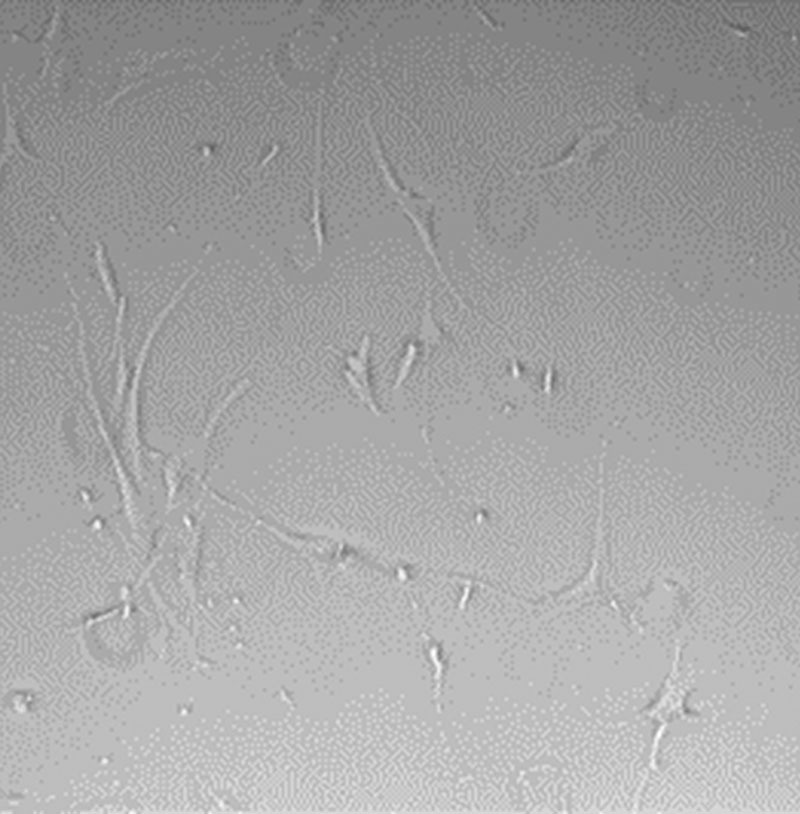
Morphology of the patient’s fibroblast culture at 96 hours. Culture medium: DMEM.

The latter evidence raises the question whether the unpredictable complications reported in elective plastic surgery procedures that are not attributable to surgical misconduct or patient’s comorbidities and/or risk factors might be related to an unexplored intrinsic cell torpidity that would be worth investigating with genetic and/or metabolic tests. Indeed, although a preliminary preoperative in vitro screening is not feasible at the moment in all the patients undergoing elective plastic surgery, an ex post study investigating such a peculiar cellular behavior might be proposed in selected cases to properly address or rebalance the respective patient/physician liabilities within the context of medico-legal litigation.

## Supplementary Material
