# Supplementary figures and images for: Unrecognized Cell Torpidity as a Risk Factor in Elective Plastic Surgery
Source: Plast Reconstr Surg Glob Open. 2018 Mar 12;6(3):e1727. doi: 10.1097/GOX.0000000000001727 (PMC5908505; doi:10.1097/GOX.0000000000001727)

**24h**

**72h**

**120h**

**Patient**

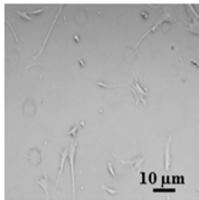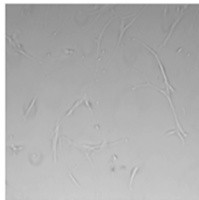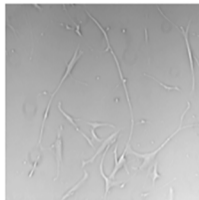

**Control**

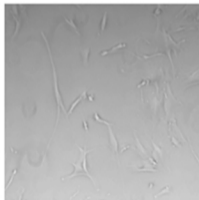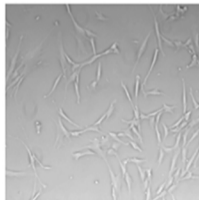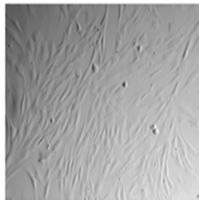

Supplement: Supplementary file 1 [file gox-6-e1727-s001.pdf]
